# Spatiotemporal clusters of HIV/AIDS infections caused by drug use and heterosexual contact in Ruili city, China 1989–2016

**DOI:** 10.1186/s12879-019-4568-0

**Published:** 2019-10-30

**Authors:** Li Jiang, Zhoulin Li, Jin Huang, Bang Liu, Yingbo Yang, Lanzhu Lin, Chengbo Wang, Ximei Xie, Xia Peng, Wen Xu, Hong Li

**Affiliations:** 1 The First Affiliated Hospital of Guizhou University of Traditional Chinese Medicine, Guiyang, Guizhou People’s Republic of China; 2 Ruili City Center for Disease Control and Prevention, Ruili, Yunnan People’s Republic of China; 3 University of Kunming Medical Science, Kunming, Yunnan People’s Republic of China; 4 Yunnan Provincial Center for Disease Control and Prevention, 158 Dongsi street, Kunming, Yunnan 650022 People’s Republic of China

**Keywords:** China, Drug use, Heterosexual contact, HIV, Spatial analysis, Ruili

## Abstract

**Background:**

Ruili is a border city in southwest China along the heroin trafficking route. In recent decades, the city has witnessed increased in HIV transmission. The current study aims to explore the spatiotemporal trends in HIV prevalence identify and map the spatial variation and clustering of factors associated with HIV transmission through drug use and heterosexual contact transmissions at the village level from 1989 through 2016.

**Methods:**

Geographic information system-based spatiotemporal analyses, including global and local spatial autocorrelation analyses and space-time scanning statistics, were applied to detect the location and extent of HIV/AIDS high-risk areas.

**Results:**

Drug use and heterosexual contact were identified as the major transmission routes causing infection in Ruili. Results of global spatial analysis showed significant clustering throughout the city caused by transmission via drug use in the early phase of the epidemic and transmission via heterosexual contact in the late phase of the epidemic during the study period. Hotspots of transmission from drug use were randomly distributed throughout the city. However, the hotspots of transmission by heterosexual contact were located in the central area only around the Jiegao China-Myanmar land port. Space-time scanning showed that transmission from drug use clustered in the southwest area between 1989 and 1990, while transmission by heterosexual contact clustered in the central area between 2004 and 2014.

**Conclusions:**

Heterosexual contact has become the dominant mode of transmission. Interventions should focus on highly clustered area where is around the Jiegao land port.

## Background

Since 1989, human immunodeficiency virus/acquired immune deficiency syndrome (HIV/AIDS) has been detected among people who inject drugs in Ruili, which is a China-Myanmar border city of Dehong Prefecture, Yunnan Province. The infectious disease has spread rapidly across the country through population movement across the Myanmar-Yunnan border into Ruili and from Yunnan to other Chinese provinces [1]. In recent decades, Ruili has suffered threats from the HIV/AIDS epidemic. The major risk originated from Myanmar, which is one of the largest producers of heroin worldwide [2]. Cheap prices and highly permeable borders make heroin easily accessible to regional residents through both Myanmar-to-China drug trafficking and Chinese residents crossing over into Myanmar to purchase and use drugs. Large numbers of injecting drug users are active in this region and are associated with the cross-border transmission of HIV [3]. On the other hand, as the largest jade trading market in China with rapid economic development and a comprehensive social environment, Ruili attracts a large number of cross-border populations from both sides. In 2014, approximately 17 million people were estimated to cross the entry and exit land ports in Ruili [4]. The large migrant populations are likely to be a large reservoir for the continuous spread of HIV.

The Chinese government and relative health institutes have implemented several measures regarding HIV prevention and control [5]. Condom promotion, needle exchange, and methadone maintenance programs and mother-to-child transmission prevention services have decreased the HIV incidence rate among drug users from 5.19 to 0.97% and among sex workers from 0.79 to 0.06%, and mother-to-child transmission rates have decreased from 8.8 to 3.1% in Yunnan from 2005 to 2016 [6]. Furthermore, these intervention programs also covered non-Chinese people who had residence, employment permits or health certificates in Yunnan. Until 2016, nearly 70,000 immigrants were offered free HIV testing; 10,198 HIV cases were identified and provided free antiviral treatment, and mother-to-child transmission prevention services were offered to 1106 people in the immigrant community of the province [6].

Yunnan has made great achievements in the prevention and control of HIV/AIDS; however, there is still a long way to go to achieve the Joint United Nations Programme on HIV/AIDS (UNAIDS) 90–90–90 targets for eradicating AIDS from the population by 2030 [7]. Thus, effective HIV services should be continually provided in Ruili, which is a high-risk area. Spatial cluster analysis plays a significant role in public health. It can assist in identifying areas with unusually high disease occurrence, which in turn helps to evaluate health care availability and health care operations. The identified clusters are also useful to define areas that require further investigation and potential intervention [8]. However, limited data on the geographical distribution of HIV/AIDS cluster in the area have been analyzed. In this study, epidemiological characteristics, spatial autocorrelation and spatiotemporal scan analyses for HIV/AIDS cases associated with either drug use or heterosexual contact were conducted to statistically evaluate the significance of aggregation and determine the size of the range of hot spots at the administrative village level in Ruili from 1989 through 2016. These results can be useful in informing HIV/AIDS prevention and control efforts in the area.

## Methods

### Study area

**Fig. 1:**
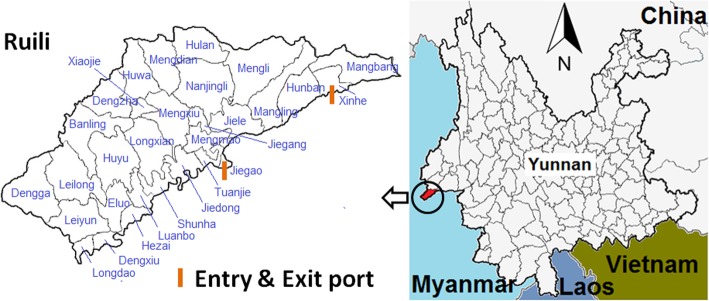
The geography of Ruili city, China. (The map layers were provided and permitted to use in the study by China Center for Disease Control and Prevention)

Ruili is the southwestern-most city in China, has an area of 1020 km^2^ and shares a 169.8 km border with Myanmar. Twenty out of a total of 30 administrative villages in the city are located on the border, with 2 border land ports at Jiegao and Xinhe village (Fig. 1). The population of the city is approximately 210,000, of which nearly 60% were reported as ethnic minorities in 2017. Due to environmental continuity, and closed cultural, historical, and linguistic ties, cross-border marriage and trade are common in the area [9].

### Data collection and management

In 1985, the HIV/AIDS case reporting system was established in China. All medical institutions, institutions for disease prevention and control, blood donation and supply organizations report HIV/AIDS cases meeting the diagnostic criteria directly to the reporting system. HIV-positive cases are considered on the basis of blood sample screening by enzyme-linked immunosorbent assays (ELISA) and confirmed by Western blot (WB), radioimmunoprecipitation (RIPA) and immunofluorescence assays (IFA). Case’s information was collected by face-to-face interviews using a national standard questionnaire at HIV/AIDS confirmatory laboratory. The high-risk behaviors recorded for all subjects were drug use, heterosexual or homosexual contact, blood products access, surgery exposure, and mother-infant transmission. Possible transmission route for each case was assessed by epidemiologist at the confirmatory laboratory.

This study involved HIV/AIDS patients who were Chinese nationals, Ruili residents, and who had a detailed address at the administrative village level recorded in the reporting system from 1989 through 2016. HIV/AIDS referred to the presence of either HIV infection, the development of AIDS, or both at the time of reporting.

### General epidemiological analysis

Case information was entered into the R program (version 3.2.1) for data exploration and analysis. The time trend was described by year. Demographic characteristics, including age, gender and occupation are presented in graphs. The annual average case prevalence rate by administrative village was explored in MapInfo (version 15, serial number: MINWCA 1500000240).

### Cluster analysis

The clusters of HIV/AIDS cases infected by either drug use or heterosexual contact were evaluated by global spatial autocorrelations, local spatial autocorrelations, and space-time scanning analyses, respectively. Population data were derived from the Information Management Section of the Yunnan Center for Disease Control and Prevention. The population of each village was employed as denominator in the analyses for HIV/AIDS cases associated with either drug use or heterosexual contact.

Global spatial autocorrelations were employed to assess the spatial distribution patterns (cluster/disperse/random) of HIV/AIDS cases associated with either drug use or heterosexuality throughout Ruili city. Values for Moran’s Index (I) close to − 1 or + 1 indicated a strong positive or negative spatial autocorrelation, respectively. When the *P*-value was lower than 0.05 in the Z test for the value, the pattern of distribution was considered to be clustered. Otherwise, the pattern was considered to be random.

Local spatial autocorrelations were used to identify clusters at the village level, which helped to understand heterogeneities within the global pattern or driving the overall clustering pattern. Local indicators of spatial autocorrelation (LISA) were examined by the Z-test. When the P-value was lower than 0.05, a local autocorrelation existed. The association patterns were divided into four categories: High-High, High-Low, Low-Low, and Low-High. Both the global and local spatial autocorrelations were performed using GeoDa software (version 1.4.6) [10].

Spatial autocorrelation analysis can determine the spatial heterogeneity of a disease; however, it has limited function in detecting the specific aggregation range of the disease and its clustering over time. A discrete Poisson model was employed to detect the distribution of HIV/AIDS clusters over space and time. The method scanned areas of high HIV incidence with a time allocation of 1 year, a maximum size of a spatial cluster equal to 50% of the at-risk population, a maximum size of a temporal cluster equal to 50% of the study period and a maximum of 999 Monte Carlo simulations. A log likelihood ratio (LLR) and relative risk (RR = observed number of cases/expected number of cases) were calculated. A cluster was considered statistically significant if the *P*-value was lower than 0.05. Space-time scanning was composed by SaTScan software (version 9.6). All of the results of the spatial analyses were visualized by using MapInfo (version 15, serial number: MINWCA 1500000240) [11].

## Results

### General epidemiological characteristics

**Fig. 2:**
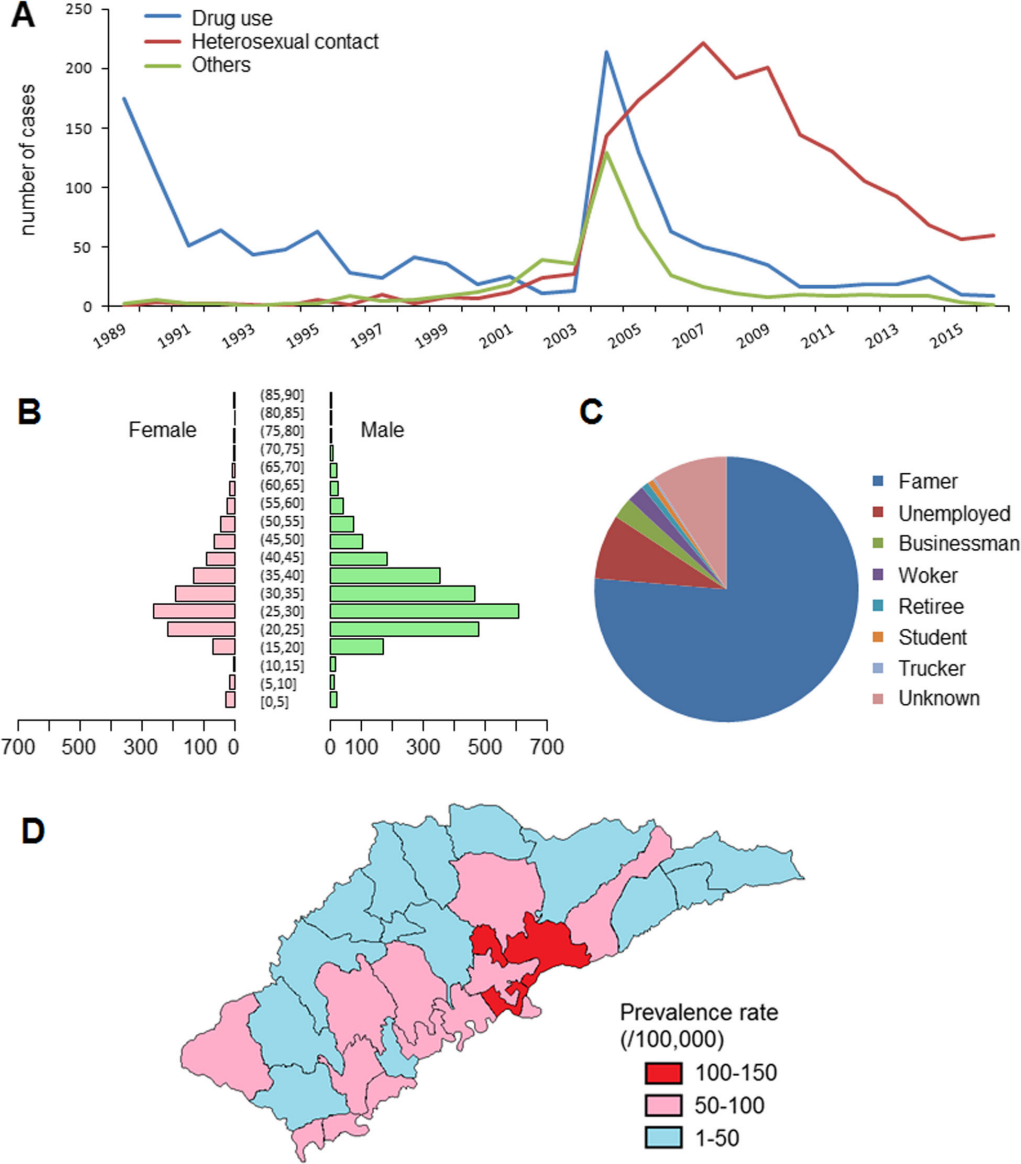
Epidemiological characteristics of HIV/AIDS in Ruili, 1989–2016. **a** Annual case number of HIV/AIDS by different transmission routes; **b** HIV/AIDS case distribution by age and gender; **c** HIV/AIDS case distribution by occupation. **d** Average HIV/AIDS annual prevalence rate (/100.000) by administrative village

During 1989–2016, 3745 HIV/AIDS cases were reported in Ruili city, with 1399 cases infected by drug use, 1889 cases infected by heterosexual contact, and 457 cases infected by other transmission routes, including homosexual contact, blood products access, surgery exposure, and mother-infant transmission. The number of cases infected by drug use was higher than that infected by heterosexual contact from 1989 to 2004, except in 2002. Then, the number of cases in the two groups reversed from 2006 to 2016 (Fig. 2a). Males accounted for 68.76% (2575/3745) of the total cases. An infection age of 25–30 years old was predominantly observed in both males and females (Fig. 2b). Farmers, unemployed persons and businessmen were the leading 3 occupation groups (Fig. 2c). The average annual prevalence rates by administrative village ranged from 5.93 to 148.80 per 100,000 individuals. Villages with high prevalence rates were mainly concentrated in the central area close to the Jiegao China-Myanmar entry and exit land port (Fig. 2d).

### Spatial cluster analysis

The yearly global spatial autocorrelation analysis of the prevalence of HIV/AIDS infections throughout Ruili showed that a significant positive spatial autocorrelation existed for cases associated with drug use during the early phase (1989 and 1991) of the study period and for cases infected by heterosexual contact during 2000, 2005, 2006, and 2013–2016 (Table 1).

**Table 1:** The global spatial autocorrelation of HIV /AIDS by drug use and heterosexual contact transmission routes in Ruili, 1989–2016

Year	Transmission route					
	Drug use			Heterosexual contact		
	Moran *I*	Z-value	*P*-value	Moran *I*	Z-value	*P*-value
1989	0.45	2.54	0.02	–	–	–
1990	0.22	1.21	0.13	0.09	0.67	0.24
1991	0.38	2.41	0.03	−0.11	−0.51	0.01
1992	−0.17	− 0.66	0.28	− 0.09	− 0.38	0.15
1993	−0.03	0.02	0.44	−0.05	−1.96	0.07
1994	−0.31	− 1.43	0.06	–	–	–
1995	0.09	0.61	0.25	−0.10	− 0.45	0.22
1996	−0.12	−0.44	0.38	−0.05	−2.09	0.11
1997	0.24	1.33	0.10	−0.12	−0.62	0.29
1998	−0.18	−0.69	0.24	−0.07	− 0.24	0.21
1999	0.06	0.44	0.34	0.01	0.23	0.32
2000	−0.19	−0.83	0.22	0.34	2.14	0.03
2001	−0.16	− 0.63	0.30	− 0.12	−0.40	0.37
2002	−0.35	−1.48	0.06	0.23	1.53	0.08
2003	0.20	1.30	0.12	−0.26	−1.42	0.02
2004	0.10	0.71	0.22	−0.13	− 0.52	0.30
2005	0.21	1.14	0.13	0.51	2.97	< 0.01
2006	0.07	0.45	0.32	0.35	2.19	0.03
2007	0.27	1.42	0.08	0.24	1.43	0.09
2008	0.09	0.59	0.28	0.05	0.49	0.30
2009	−0.05	−0.06	0.48	−0.16	−0.65	0.27
2010	0.16	1.04	0.15	0.13	0.91	0.17
2011	0.02	0.37	0.27	0.25	1.48	0.08
2012	0.17	1.05	0.15	0.13	0.96	0.18
2013	0.15	1.01	0.17	0.34	2.30	0.02
2014	−0.26	−1.17	0.12	0.30	2.35	0.03
2015	0.19	1.17	0.13	0.23	1.88	0.04
2016	−0.16	−0.83	0.14	0.38	2.57	0.02
Average	−0.02	0.01	0.49	0.46	3.00	< 0.01

**Fig. 3:**
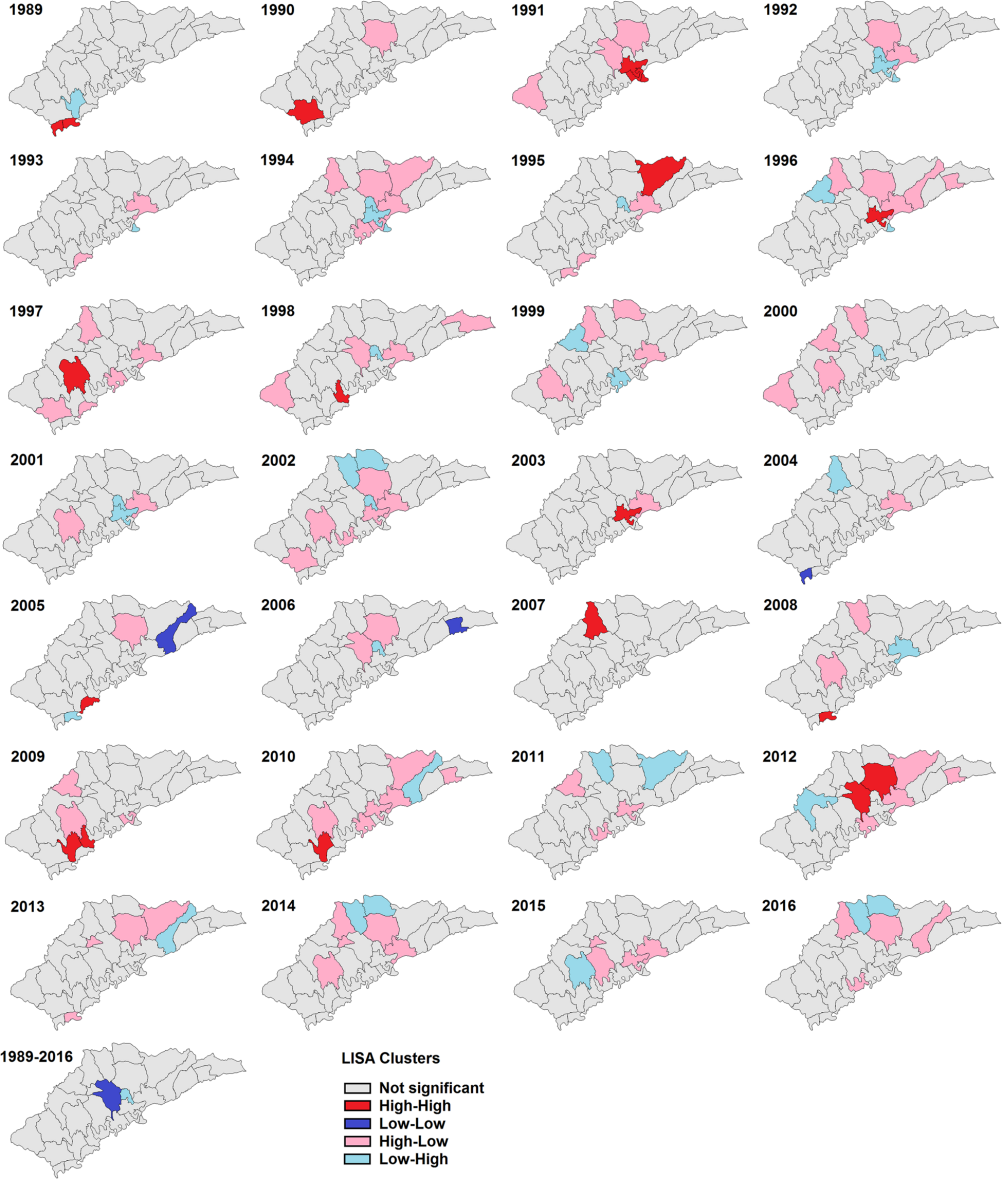
Yearly local indicators of spatial association (LISA) cluster maps for HIV/AIDS prevalence by drug use transmission in Ruili, 1989–2016

**Fig. 4:**
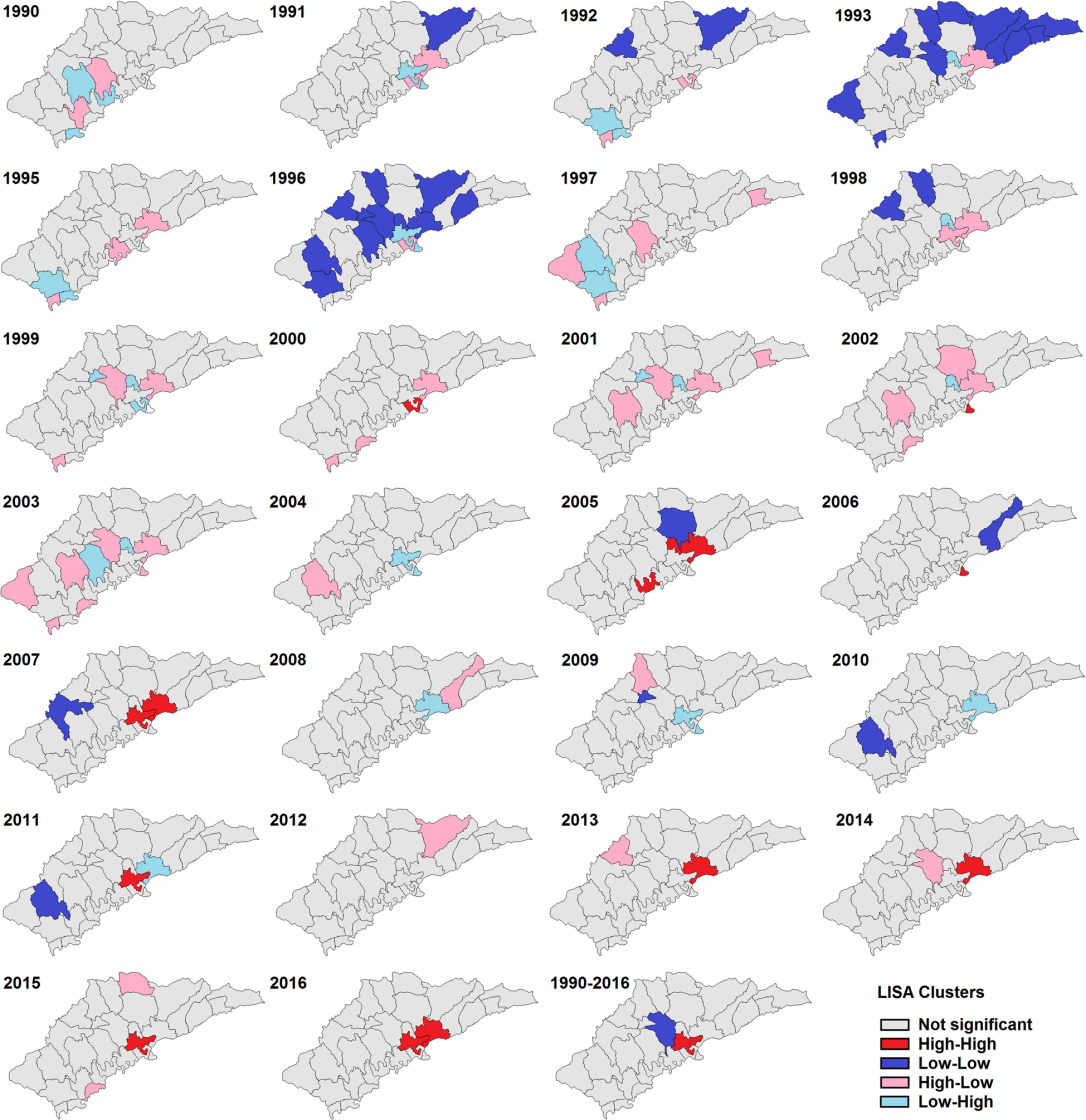
Yearly local indicators of spatial association (LISA) cluster maps for HIV/AIDS prevalence by heterosexual contact transmission in Ruili, 1989–2016

The LISA analysis identified the hot spots (High-High) of HIV/AIDS. Hot spots for cases associated with drug use were observed randomly throughout the area (Fig. 3). However, hot spots for cases associated with heterosexual contact were only located in the central area of Ruili, which is close to the Jiegao land port (Fig. 4).

**Fig. 5:**
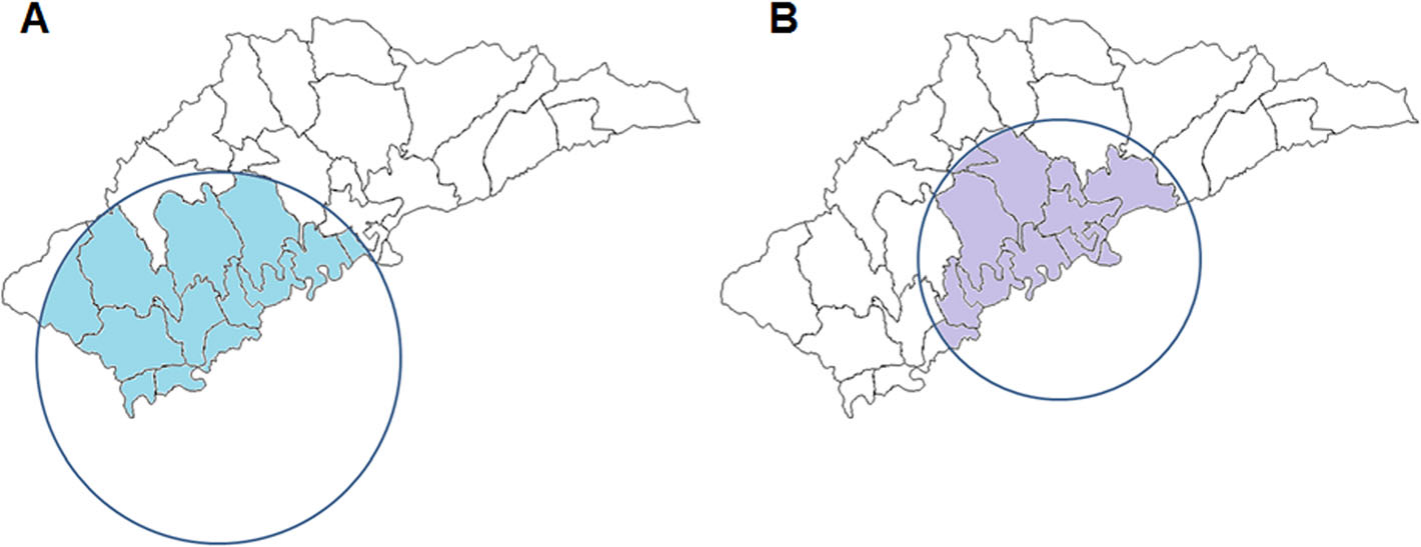
Case clusters of HIV/AIDS infection by drug use (**a**) and heterosexual contact (**b**) transmission through space-time analysis in Ruili, 1989–2016

The space-time scanning analysis identified significant clusters over different time intervals and spaces for drug use and heterosexual infection. Only one most likely cluster was detected for drug use and heterosexual infection, respectively, when the maximum spatial cluster size was set as 50% of the population at risk in the spatial window (The results were listed in Additional file 1: Table S1 and S2, when the maximum spatial cluster size was set to 40–20% of the population at risk in the spatial window). The drug use transmission cluster was detected in the southwest area between 1989 and 1990 with a radius of 17.05 km, covering 13 villages in Ruili. Compared to other villages, those identified in this cluster had a 5.67 times higher HIV risk among local people. The heterosexuality transmission cluster was identified in the central area between 2004 and 2014 with a radius of 12.85 km, covering 12 villages of Ruili, Compared to other villages, those identified in this cluster had a 5.55 times higher relative risk among local people (Table 2, Fig. 5).

**Table 2:** Spatial and temporal cluster of HIV /AIDS by drug use or heterosexual contact transmission routes in Ruili, 1989–2016

Transmission route	Potential clusters	Latitude	Longitude	Radius (km)	Time interval	Total locations	Log likelihood ratio	Relative risk	*P* value
Drug use	Cluster 1	23.894737	97.734959	17.05	1989–1990	13	196.21	5.67	< 0.01
Heterosexuality	Cluster 1	23.966129	97.834522	12.85	2004–2014	12	656.49	5.55	< 0.01

## Discussion

The present study explored the spatiotemporal features of HIV/AIDS cases since the beginning of infection detection in Ruili. Drug use and heterosexual contact were major transmission routes causing the infection spread in the area. Global spatial analyses showed significant clustering throughout the city for cases of transmission via drug use in the early phase of the outbreak and cases of transmission via heterosexual contact mainly in the late phase during the study period. The number of transmission hotspots associated with drug use was observed sporadically throughout the city. However, the heterosexual transmission hotspots were located only in the central area. Space-time scanning detected a drug use transmission cluster in the southwest area between 1989 and 1990 and a heterosexual transmission cluster in the central area between 2004 and 2014.

The transmission rate from drug use among total cases decreased in the last decade in Ruili, with a rate of 12.9% in 2016, which was much higher than the national level of 3.2% [12]. Helping to fuel the epidemic is Ruili’s positioning as the first station of Myanmar-to-China drug trafficking. Drug trafficking in the region has contributed to the high prevalence of HIV infection within the country [13]. The spatial analysis in our study showed a cluster of drug use transmission in the southwest area during the first 2 years of the study period. The HIV prevalence among Burmese injecting drug users (IDUs) in Northern Myanmar and migrant IDUs from Myanmar to Yunnan was sustained at 13.1 and 19.0%, respectively [14, 15]. Burmese IDUs in Yunnan play a pivotal role in the cross-border transmission of HIV [15]. As estimated, there were 3940 autochthonic drug users in Ruili in 2011 [16]. Due to blood exposure through drug-injection equipment, the prevalence of HIV among these drug users reached 16.4% in 2012 [17]. The needle exchange programs (NEP) and methadone maintenance treatment (MMT) program have been implemented since 2002 and 2005 in the area. It has succeeded in improving the quality of life and reducing the risk of HIV infection among heroin-dependent patients [18–20]. However, we observed hot spots distributed sporadically throughout the city after 1990. This phenomenon might reflect the risk of HIV infection among IDUs. By the end of 2014, the proportion of IDUs with AIDS receiving no MMT was approximately 60% in the area. Being female, a farmer, and of an ethnic group and having a low educational level and short diagnosed HIV infection history were risk factors for the barrier to MMT access [21]. Thus, improving MMT coverage for both Chinese and foreign nationals should be implemented immediately.

The significant decrease of HIV infection by drug use could result from the implement of the NEP and MMT. However, heterosexual contact has become the dominant mode of transmission among newly diagnosed HIV cases in the area since 2006. The trends alarmed that the HIV epidemic has transited from the high-risk populations to the general population [22]. Furthermore, the spatial analysis in our study demonstrated that a cluster of heterosexual transmission cases occurred between 2004 and 2014 without cluster either for drug use or for heterosexual transmission during the years 1991 and 2003. The phenomenon was caused by the expending the scope of HIV testing since 2004, which resulted more HIV case detections in the area. On the other hand, government has been concentrating on HIV control among drug users in the early epidemic time. However, transmission by heterosexual contact hasn’t drawn much attention during the period. Thus, HIV infections were trended to accumulate among general population and caused severe epidemic in the late time. The heterosexual contact infection clustered in the central area, with the Jiegao land port as the central radiation point. Jiegao is the largest land port in China for exports to Myanmar and shoulders a large cross-border population for work and trade [23]. The HIV infection ratio among travelers entering the area was 5.12%, which was higher than that of any of the border counties in Yunnan [24]. Furthermore, there are more than 50,000 Burmese living in Ruili alone [7]. Commercial sex is common in the city, and there was a low condom use rate in the early phase of the HIV epidemic [25]. Intervention strategies and knowledge regarding the three HIV transmission routes and condom usage can reduce the risk of HIV infection, and the condom use rate increased from 40 to 80% among sex workers [26]. However, our study detected hot spots in the area during 2013–2016. The phenomenon demonstrated that interventions and knowledge regarding condom use should be enhanced in the area. In addition, there were more than 25,000 Burmese brides living in Yunnan [27]. The HIV prevalence was 2.2% among this group of people in Tengchong city, a neighboring county of Ruili [28]. Thus, the general population with non-commercial sexual behaviors maintains a high risk of HIV infection. Effective interventions including health education and health services should be expanded in Ruili city.

There were several limitations to this study. It only included cases reported in the surveillance system and most likely underrepresented the extent of HIV/AIDS prevalence. Moreover, unidentified cases (without a detailed address) were excluded from the study. The denominator population was population of each village, rather than exact population at exposure risk. These might introduce some bias in the study. However, due to the extensive ongoing monitoring system across Ruili, the bias would be minimized. Furthermore, the study setting was involved a city where has 30 administrative villages. Although the circular scan window detected large clusters, narrower cluster in geography was necessary for the disease control in a city level. Thus, it requires more detailed geographic information and stratification in the further study.

## Conclusion

In conclusion, HIV/AIDS is a severe public health problem in Ruili. Heterosexual contact has become the dominant mode of transmission. Interventions in health education and health services should focus on highly clustered areas around the Jiegao land port. Moreover, drug use presented sporadically throughout the city; therefore, MMT coverage needs to expand countywide.

## Supplementary information

**Additional file 1: Table S1.** Spatial and temporal cluster of HIV /AIDS by different drug use transmission routes in Ruili, 1989–2016, using different percent of the population at risk. **Table S2.** Spatial and temporal cluster of HIV /AIDS by different heterosexual contact transmission routes in Ruili, 1989–2016, using different percent of the population at risk.

## Data Availability

The datasets used and analyzed during the current study are available from the corresponding author on reasonable request.

## Abbreviations

AIDS: Acquired immune deficiency syndrome
HIV: Human immunodeficiency virus
IDU: Injecting drug user
LISA: Local indicators of spatial autocorrelation
MMT: Methadone maintenance treatment
