# Supplementary material for: Spatiotemporal clusters of HIV/AIDS infections caused by drug use and heterosexual contact in Ruili city, China 1989–2016
Source: BMC Infect Dis. 2019 Oct 30;19:925. doi: 10.1186/s12879-019-4568-0 (PMC6822478; doi:10.1186/s12879-019-4568-0)
Supplement: Supplementary file 1 — Additional file 1: Table S1. Spatial and temporal cluster of HIV /AIDS by different drug use transmission routes in Ruili, 1989–2016, using different percent of the population at risk. Table S2. Spatial and temporal cluster of HIV /AIDS by different heterosexual contact transmission routes in Ruili, 1989–2016, using different percent of the population at risk. [file 12879_2019_4568_MOESM1_ESM.docx]

**Table S1** Spatial and temporal cluster of HIV /AIDS by different drug use transmission routes in Ruili, 1989-2016, using different percent of the population at risk

| Percent of the population at risk | Cluster type | Radius (km) | Time interval | Total locations | Log likelihood ratio | Relative risk | *P* value |
| --- | --- | --- | --- | --- | --- | --- | --- |
| 50% | Most likely | 17.05 | 1989-90 | 13 | 196.21 | 5.67 | <0.01 |
| 40% | Most likely | 14.76 | 1989-90 | 11 | 179.76 | 6.09 | <0.01 |
|  | Secondary | 13.84 | 2004-05 | 10 | 100.50 | 4.25 | <0.01 |
| 30% | Most likely | 8.78 | 1989-90 | 8 | 179.48 | 6.98 | <0.01 |
|  | Secondary | 13.08 | 2004-05 | 8 | 91.90 | 4.62 | <0.01 |
|  | 2^nd^ Secondary | 3.04 | 1989-96 | 2 | 60.01 | 3.24 | <0.01 |
| 20% | Most likely | 3.30 | 1989-90 | 3 | 173.80 | 11.26 | <0.01 |
|  | Secondary | 9.89 | 2004-04 | 5 | 89.09 | 8.19 | <0.01 |
|  | 2^nd^ Secondary | 11.61 | 2004-04 | 8 | 66.52 | 7.15 | <0.01 |
|  | 3^rd^ Secondary | 3.04 | 1989-96 | 2 | 60.01 | 3.24 | <0.01 |

**Table S2** Spatial and temporal cluster of HIV /AIDS by different heterosexual contact transmission routes in Ruili, 1989-2016, using different percent of the population at risk

| Percent of the population at risk | Cluster type | Radius (km) | Time interval | Total locations | Log likelihood ratio | Relative risk | *P* value |
| --- | --- | --- | --- | --- | --- | --- | --- |
| 50% | Most likely | 12.85 | 2004-14 | 12 | 656.49 | 5.55 | <0.01 |
| 40% | Most likely | 12.80 | 2004-14 | 9 | 653.44 | 5.75 | <0.01 |
| 30% | Most likely | 4.37 | 2004-16 | 4 | 497.12 | 5.43 | <0.01 |
|  | Secondary | 8.78 | 2004-11 | 7 | 161.53 | 3.12 | <0.01 |
| 20% | Most likely | 3.81 | 2004-16 | 4 | 438.40 | 5.09 | <0.01 |
|  | Secondary | 5.52 | 2004-11 | 5 | 120.27 | 3.08 | <0.01 |
|  | 2^nd^ Secondary | 6.58 | 2004-10 | 4 | 94.26 | 3.18 | <0.01 |
|  | 3^rd^ Secondary | 11.14 | 2005-09 | 6 | 23.77 | 2.15 | <0.01 |
